# Phyloproteomic and functional analyses do not support a split in the genus *Borrelia* (phylum Spirochaetes)

**DOI:** 10.1186/s12862-019-1379-2

**Published:** 2019-02-13

**Authors:** Agustín Estrada-Peña, Alejandro Cabezas-Cruz

**Affiliations:** 1 Department of Animal Pathology, Faculty of Veterinary Medicine, Miguel Servet, 177, 50013 Zaragoza, Spain; 2 UMR BIPAR, INRA, ANSES, Ecole Nationale Vétérinaire d’Alfort, Université Paris-Est, 94700 Maisons-Alfort, France

**Keywords:** *Borrelia*, *Borreliella*, Phyloproteomics, Functional analysis, Unsupported split

## Abstract

**Background:**

The evolutionary history of a species is frequently derived from molecular sequences, and the resulting phylogenetic trees do not include explicit functional information. Here, we aimed to assess the functional relationships among bacteria in the Spirochaetes phylum, based on the biological processes of 42,489 proteins in reference proteomes of 34 Spirochaetes species. We tested the hypothesis that the species in the genus *Borrelia* might be sufficiently different to warrant splitting them into two separate genera.

**Results:**

A detrended canonical analysis demonstrated that the presence/absence of biological processes among selected bacteria contained a strong phylogenetic signal, which did not separate species of *Borrelia*. We examined the ten biological processes in which most proteins were involved consistently. This analysis demonstrated that species in *Borrelia* were more similar to each other than to free-life species (*Sediminispirochaeta*, *Spirochaeta*, *Sphaerochaeta*) or to pathogenic species without vectors (*Leptospira*, *Treponema, Brachyspira*), which are highly divergent. A dendrogram based on the presence/absence of proteins in the reference proteomes demonstrated that distances between species of the same genus among free-life or pathogenic non-vector species were higher than the distances between the 19 species (27 strains) of *Borrelia*. A phyloproteomic network supported the close functional association between species of *Borrelia*. In the proteome of 27 strains of *Borrelia*, only a few proteins had evolved separately, in the relapsing fever and Lyme borreliosis groups. The most prominent *Borrelia* proteins and processes were a subset of those also found in free-living and non-vectored pathogenic species. In addition, the functional innovation (i.e., unique biological processes or proteins) of *Borrelia* was very low, compared to other genera of Spirochaetes.

**Conclusions:**

We found only marginal functional differences among *Borrelia* species. Phyloproteomic networks that included all pairwise combinations between species, proteins, and processes were more effective than other methods for evaluating the evolutionary relationships among taxa. With the limitations of data availability, our results did not support a split of the arthropod-transmitted spirochaetes into the proposed genera, *Borrelia* and *Borreliella*.

**Electronic supplementary material:**

The online version of this article (10.1186/s12862-019-1379-2) contains supplementary material, which is available to authorized users.

## Background

The phylum Spirochaetes is a group of widely distributed bacteria that includes saprophytic and parasitic species, which can affect both human and animal health. Spirochaetes includes the families Spirochaetaceae, Brevinemataceae, Brachyspiraceae, Leptospiraceae, and Borreliaceae. Based on sequence reconstruction and phylogenetic analysis, it has been proposed that some of these families should be elevated to a higher taxonomic status [1]. There are four clinically important genera in this phylum, whose species are the etiological agents of major diseases*.* One is *Treponema*, of which *T. pallidum* is the causative agent of syphilis, a sexually transmitted disease distributed worldwide [2]. Other members of the genus *Treponema* play important roles in periodontal diseases [3]. Species in the genus *Borrelia* includes 37 taxa, which are commonly separated into two main clades. One clade includes the etiological agents of the Lyme borreliosis group, which are frequently referred to as the *Borrelia burgdorferi* sensu lato complex; these are transmitted by ticks of the *Ixodes ricinus* complex. The second clade contains the causative agents of the relapsing fever group; these species are transmitted by a variety of ticks, except for *B. recurrentis*, which uses lice as the vector [4–9]. Two other genera, *Leptospira* and *Brachyspira*, contain the agents of leptospirosis and intestinal spirochaetosis, respectively [10, 11]. Other taxa in the phylum, like those in the genera *Spirochaeta*, *Sphaerochaeta*, and *Sediminispirochaeta*, contain free-living species.

Whole genome sequences, which are becoming increasingly available in public databases can be used to reconstruct the evolutionary history of the Spirochaetes. It was proposed that the genus *Borrelia* should be split in two, *Borrelia* and *Borreliella*, based on the evolutionary history derived from the identification of conserved signature insertions and/or deletions (indels) that are present exclusively in protein sequences of these organisms [1]. Other authors supported the split of both genera with analyses of proteins that are conserved only in the family Borreliaceae [12, 13]. However, provided empirical proofs supported the inadequacy of a genus split [14]. Evolutionary, ecological, and geological reconstructions of the putative scenario of speciation of ticks and the genus *Borrelia* [15] have provided further evidence in favor of reconsidering the proposed split. Available data support that the association of *Borrelia* with ticks occurred before the major split between the tick families Argasidae–Ixodidae (dated some 230–290 Mya) resulting in relapsing fever (Rf) species being restricted to Argasidae and few associated with Ixodidae. Further key events produced the diversification of the Lyme borreliosis (Lb) species and the group of species associated to Reptilia. We hypothesized that the evolutionary pressures on Rf were low, since speciation processes seem to be associated with the geographical isolation of the transmitting ticks and not with host diversity. In contrast, Lb species circulate in networks of dozens of tick species and hundreds of vertebrate species. This greater variety of hosts may have been associated with high evolutionary pressure which in turn resulted in a large speciation of Lb [15]. Few data are available about the Reptilia-associated *Borrelia* spp. to draw conclusions, but they seem to represent a parallel lineage between Rf and Lb.

We hypothesized that a complete, functional, comparative framework might provide additional details of the phylogenetic relationships among selected species of the phylum Spirochaetes. This framework could facilitate comparisons of functional differences between proteomes, as proposed previously [16], reconstructing trees of life based on the functions of proteins. We aimed to challenge the hypothesis that the *Borrelia* genus should be split in a context that included other taxa of the phylum, which allowed us to assess the degree of relatedness among the species included in the Borreliaceae family. We analyzed functional differences by comparing biological processes (BPs) and building phylogenetic trees, based on pairwise comparisons of the patterns of the presence/absence of proteins. Moreover, we implemented a novel approach, known as the phyloproteomic network, which allowed functional comparisons within an evolutionary context. Phyloproteomic networks are constructed based on information about the presence/absence of orthologous proteins [17]. We obtained this information from the manually curated, revised, annotated reference proteomes of 34 species and 8 strains of the phylum Spirochaetes. These reference proteomes included proteins found in free-living taxa, pathogens without vectors, and species adapted to transmission by arthropods. We expected these procedures to provide an explanation for the genome reduction known to occur in this group of bacteria. We also expected to obtain additional data that could support or reject the proposed split of *Borrelia* into two different genera.

## Results

### Species in Borrelia display the lowest functional diversity of biological processes

**Fig. 1:**
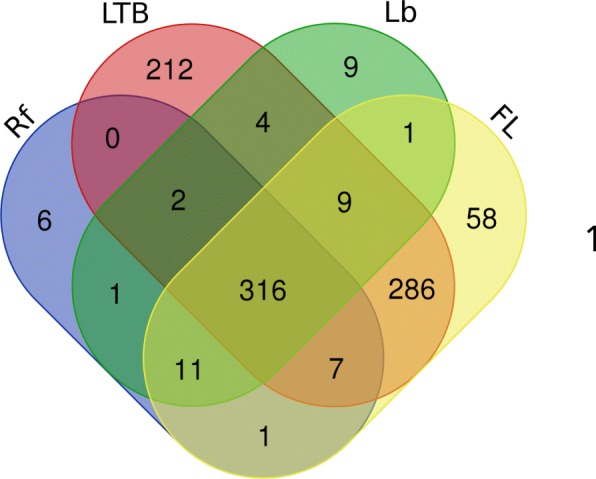
Venn diagram displays shared biological processes among different bacteria species. The numbers indicate the numbers of cell processes that are unique (no overlap) or shared (intersecting areas) by the different groups of spirochaetes included in this study. Rf: Relapsing fever group; LTB: *Leptospira* spp., *Treponema* spp., and *Brachyspira* spp.; Lb: Lyme borreliosis group; FL: free-living species, including the genera *Spirochaeta*, *Sphaerochaeta,* and *Sediminispirochaeta*

Data from 41 reference proteomes (33 species plus 8 strains) were retrieved from InterProt and linked to annotations in the Gene Ontology (GO) website (see Table 1 for the complete details about the selected proteomes). The selection of proteomes was based on: (i) species diversity (to have the greatest range of species for comparisons) and completeness (i.e. the reference proteome is the most complete one with as many annotated proteins as possible). These data included a total of 42,489 proteins, which were annotated in 924 BPs. Each BP was represented by a variable number of proteins. We constructed a Venn diagram to compare the total number of BPs identified in all the taxa included in this study (Fig. 1). The complete list of proteins per BP is available in Additional file 1. All the Spirochaetes included in this study shared a core of 316 BPs. Among these groups, *Leptospira-Treponema-Brachyspira* (LTB) and free-living species (FL), shared the highest number of BPs (i.e., 286). The LTB group had the highest number of unique BPs (i.e., 212) compared to FL, Lyme Borreliosis (Lb), and relapsing fever *Borrelia* (Rf), which had 58, 9, and 6 unique BPs, respectively.

**Table 1:** The list of species and strains included in this study, with reference to the identification number of the proteome and the organism in the UniProt database, as well as the number of proteins annotated

Organism	Group	Proteome ID	Organism ID	Protein count
*Borrelia afzelii* (strain PKo)	Lb	UP000005216	390,236	1394
*Borrelia anserina*	Rf	UP000019262	1,313,293	855
*Borrelia bavariensis* (Strain: ATCC BAA-2496 / DSM 23469 / PBi)	Lb	UP00000276	290,434	1262
*Borrelia bissettiae* (Strain: CO275)	Lb	UP000183624	64,897	1794
*Borrelia bissettiae* (Strain: DN127)	Lb	UP000001634	521,010	1403
*Borrelia burgdorferi* (strain ATCC 35210 / B31 / CIP 102532 / DSM 4680)	Lb	UP000001807	224,326	1290
*Borrelia burgdorferi* (strain ZS7)	Lb	UP000006901	445,985	1223
*Borrelia burgdorferi* 64b	Lb	UP000006162	498,740	1518
*Borrelia coriaceae* Co53	Rf	UP000019330	1,313,292	895
*Borrelia crocidurae* (strain Achema)	Rf	UP000005212	1,155,096	1469
*Borrelia crocidurae* DOU	Rf	UP000000612	1,293,575	845
*Borrelia duttonii* (strain Ly)	Rf	UP000000611	412,419	1287
*Borrelia duttonii* CR2A	Rf	UP000019148	1,432,657	1995
*Borrelia garinii* PBr	Lb	UP000006103	498,743	1346
*Borrelia hermsii* (Strain: DAH-2E7)	Rf	UP000075229	140	1230
*Borrelia hermsii* MTW	Rf	UP000019324	1,313,291	840
*Borrelia mayonii* (Strain: MN14–1539)	Lb	UP000185492	1,674,146	1131
*Borrelia miyamotoi* (Strain: CT13–2396)	Rf	UP000176410	47,466	1079
*Borrelia miyamotoi* FR64b	Rf	UP000019332	1,292,392	874
*Borrelia parkeri* SLO	Rf	UP000019331	1,313,294	873
*Borrelia recurrentis* (strain A1)	Rf	UP000000612	412,418	977
*Borrelia turicatae* (strain 91E135)	Rf	UP000019262	1,313,293	855
*Borreliella chilensis* (Strain: VA1)	Lb	UP000030940	1,245,910	898
*Borreliella finlandensis* (Strain: SV1)	Lb	UP000006166	498,741	1300
*Borreliella spielmanii* A14S	Lb	UP000003481	498,742	1297
*Borreliella valaisiana* VS116	Lb	UP000006163	445,987	1349
*Brachyspira hyodysenteriae* (strain ATCC 49526 / WA1)	LTB	UP000001803	565,034	2642
*Brachyspira pilosicoli* B2904	LTB	UP000007346	1,133,568	2637
*Leptospira biflexa* serovar Patoc (strain Patoc 1 / ATCC 23582 / Paris)	LTB	UP000001847	456,481	3723
*Leptospira borgpetersenii* str. 200,701,203	LTB	UP000011783	1,193,007	4773
*Leptospira interrogans* serovar Lai	LTB	UP000001408	57,678	3676
*Sediminispirochaeta smaragdinae* (strain DSM 11293 / JCM 15392 / SEBR 4228)	FL	UP000002318	573,413	4211
*Sphaerochaeta coccoides* (strain ATCC BAA-1237 / DSM 17374 / SPN1)	FL	UP000007939	760,011	1819
*Sphaerochaeta globosa* (strain ATCC BAA-1886 / DSM 22777 / Buddy)	FL	UP000008466	158,189	3007
*Sphaerochaeta pleomorpha* (strain ATCC BAA-1885 / DSM 22778 / Grapes)	FL	UP000005632	158,190	3150
*Spirochaeta africana* (strain ATCC 700263 / DSM 8902 / Z-7692)	FL	UP000007383	889,378	2766
*Spirochaeta lutea*	FL	UP000029692	1,480,694	2285
*Spirochaeta thermophila* (strain ATCC 49972 / DSM 6192 / RI 19.B1)	FL	UP000001296	665,571	2199
*Treponema denticola* (strain ATCC 35405 / CIP 103919 / DSM 14222)	LTB	UP000008212	243,275	2753
*Treponema maltophilum* ATCC 51939	LTB	UP000014541	1,125,699	2287
*Treponema pallidum* (strain Nichols)	LTB	UP000000811	243,276	1028

The organisms are alphabetically sorted and are included according to its standard denomination in UniProt. The column “group” indicates the four main groups of taxa considered in this study: *Rf* relapsing fever species of *Borrelia*, *Lb* Lyme borreliosis group of *Borrelia* spp., *FL* free-life species, *LTB* parasitic species without vector

**Fig. 2:**
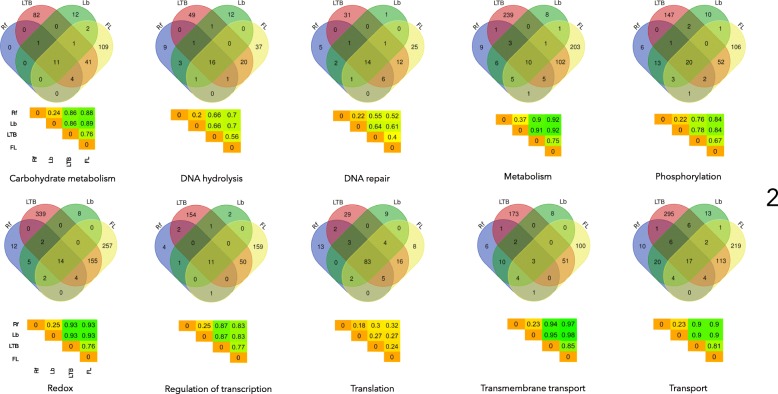
Venn diagrams display shared proteins in the ten most important biological processes (BP) in the species of Spirochaetes targeted in this study. The numbers indicate the mean number of proteins per species involved in each BP. Heat maps included below the Venn diagram of each BP show the Sorensen’s dissimilarity index between the proteins involved in a given process for the different species of bacteria. Rf: Relapsing fever group; LTB: *Leptospira* spp., *Treponema* spp., and *Brachyspira* spp.; Lb: Lyme borreliosis group; FL: free-living species, including the genera *Spirochaeta*, *Sphaerochaeta,* and *Sediminispirochaeta*. The total number of proteins involved in each process is as follows: Carbohydrate metabolism: 263, DNA hydrolysis: 150, DNA repair: 99, Metabolism: 594, Phosphorylation: 358, Redox: 798, Regulation of transcription: 385, Translation: 174, Transmembrane transport: 359, Transport: 711

The BPs were ranked according the number of proteins involved. We then examined protein sharing among the 10 highest-ranked BPs, including: carbohydrate metabolism, DNA hydrolysis, DNA repair, metabolism, phosphorylation, redox, regulation of transcription, translation, transmembrane transport, and transport (Fig. 2). We calculated the index of dissimilarity (ID) for each BP, based on the patterns of protein presence/absence. Both LTB and FL species had the highest number of unique proteins involved in these processes. LTB and FL also shared the highest number of proteins for each process. In four BPs (metabolism, redox, transmembrane transport, and transport), LTB and FL had the highest number of unique proteins and the lowest similarities with species in other groups. Rf and Lb had the lowest number of unique proteins involved in these BPs; indeed, they shared a significant number of proteins between each other and with species in the LTB and FL groups. In all comparisons of BPs, the LTB species were always more similar to FL than to the species in the Rf or Lb groups. Likewise, the Rf and Lb species showed more similarity to each other than to the FL and LTB species. Some BPs, like ‘DNA repair’ (maximum ID 0.52) and ‘translation’ (maximum ID 0.32), were highly conserved (i.e., a high number of proteins involved in these BPs were shared by all species). In fact, 83 proteins (out of 174) involved in these BPs were shared among all the species. Interestingly, ‘metabolism’ and ‘transport’ were the two processes for which *Borrelia* spp. were highly similar (low values of ID) and displayed highest differences with FL and LTB.

### *Multivariate statistics based on BPs show marginal functional difference between* Borrelia *species*

**Fig. 3:**
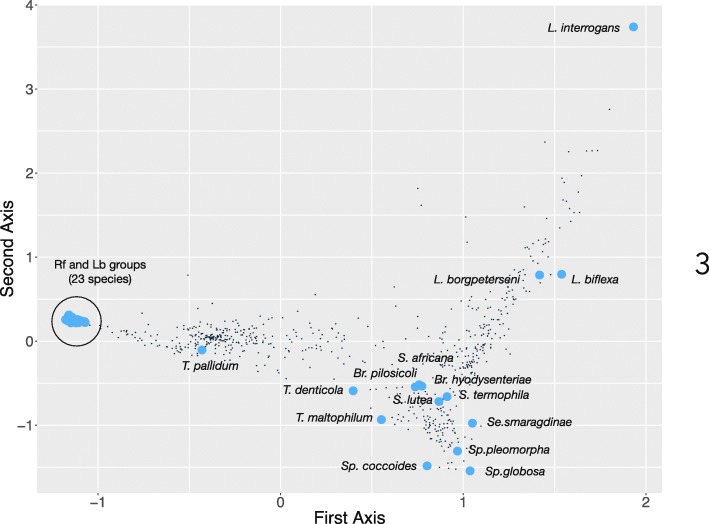
Detrended Canonical Analysis plot of all biological processes (BPs) found in 41 species of the phylum Spirochaetes displays the similarities between species or strains. Each small black point (unlabeled) represents a BP. The blue points are the species or strains of spirochaetes, placed along the two first axes of the DCA. At the scale shown, the species in the groups Rf and Lb (circled) cannot be separated into individual points; instead, they are all tightly clustered (grouped inside the black circle) and separated from the other taxa. Abbreviations: L: *Leptospira*, T: *Treponema*, Br: *Brachyspira*, Se: *Sediminispirochaeta*, Sp: *Sphaerochaeta*, S: *Spirochaeta*

The data on the presence and absence of BPs in species/strains were used to produce a Detrended Canonical Analysis (DCA). The DCA was used to evaluate the clustering of taxa along the axes of variability in shared BPs (Fig. 3). All species in the Rf and Lb groups clustered together (left side of the chart in Fig. 3). Species in the FL and LTB groups were widely spread out in the chart, and well separated from the Rf-Lb cluster. The DCA could not separate the 23 species-strains included in the Rf-Lb groups. In this two-dimensional arrangement, we found more distance between species of the same genus in FL or LTB than among any species in Rf and Lb. Moreover, the DCA showed a phylogenetically coherent view, where species from the same genus were close together and separated from species from other genera. This finding validated the DCA approach.

### Indexes based on protein presence show high relatedness between Rf and Lb species

**Fig. 4:**
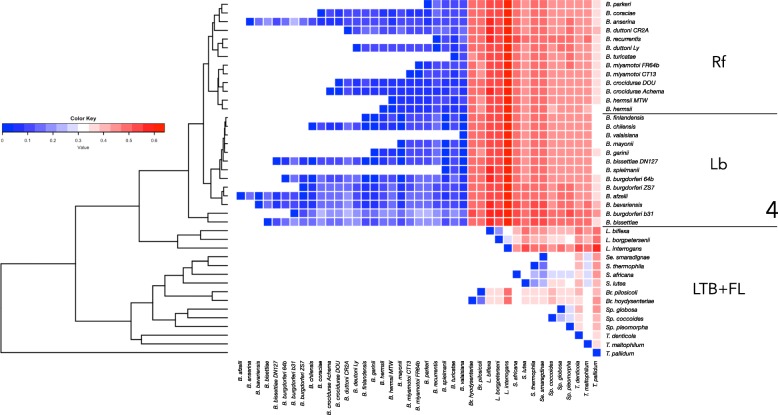
A heat map displays Jaccard’s dissimilarity indexes, calculated from the presence/absence comparison of proteins of every species in seven genera of the phylum Spirochaetes. The index of dissimilarity values are color-coded (note the color scale). Cold colors indicate low dissimilarity and warm colors represent high dissimilarity. The resulting dendrogram, based on the inverse of the Jaccard’s dissimilarity index, is inserted at left of the heat map

The presence/absence of 5604 unique proteins and 41 species-strains of the phylum were used to build a dendrogram, based on the inverse of the ID (Fig. 4). The highest similarities were consistently observed among species in the Rf-Lb group. The genera, *Sphaerochaeta*, *Spirochaeta*, and *Sediminispirochaeta*, of the FL group clustered separately from the other parasitic species. All the LTB species clustered in different branches, partly because *Brachyspira* and *Leptospira* are sister groups of Rf-Lb. It is interesting to note the low similarity of the *Treponema* species from the other species included in the dendrogram.

This functional dendrogram pointed to a higher similarity within species of the Lb group than among the species of the Rf group. Of note, the similarity based on protein presence/absence was lower between species of well-supported genera (i.e., *Brachyspira* and *Leptospira*) than the similarity between species of the Rf and Lb groups (see the overlapping LTB and FL groups in Fig. 4). The similarity among the complete set of Rf and Lb species was higher than between any other species within the other genera. The only exception was the genus *Brachyspira*; however, we studied only two species in that genus, which probably affected the observed results.

### Phyloproteomic networks reveal proteome reduction in Rf and Lb

**Fig. 5:**
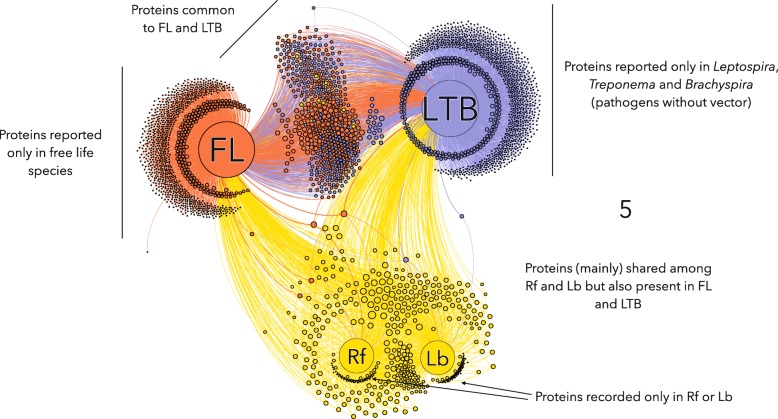
Phyloproteomic network shows the shared proteins in the 41 species and strains of Spirochaetes. The main four groups of species are Rf: Recurrent fever group; Lb: Lyme borreliosis group; FL: Free life species of the genera *Sediminispirochaeta*, *Spirochaeta* and *Sphaerochaeta*; and LTB: *Leptospira*, *Treponema*, *Brachyspira*. The small, unlabeled circles represent proteins, and the four large, labeled circles represent the indicated species. The proteins are either clustered around a group of species (unique proteins) or they are linked (shared) with other groups. The links and circles located between groups are color-coded according to the cluster of origin (blue: FL; red: LTB; yellow: Rf and Lb). The size of the circle indicates the weighted degree of importance in the network, which is related to the number of times the protein is shared by the species of Spirochaetes; the smallest points indicate that the protein was only recorded in one group, the largest circles indicate that the protein is shared by all four groups of species

We built a phyloproteomic network that comprised the four main groups of taxa (Rf, Lb, LTB and FL) and the complete set of proteins unambiguously identified in every reference proteome (Fig. 5). We built a network, because it can show relationships between nodes (which may be taxa, proteins, or BPs) and provide the relative importance of each node in the resulting associations between species, their proteins, and the associated BPs. Our network pinpointed two main features of the proteomic relationships between the four groups of taxa: (*i*) a large set of proteins was shared exclusively by FL and LTB, and both groups evolved a large number of proteins exclusive from each other; (*ii*) a set of proteins was shared by every group of taxa, and these were the most prominent proteins (in terms of relative importance) in the proteomes of Rf and Lb; and (*iii*) both Rf and Lb evolved a small number of unique proteins that were exclusively and separately found in either Rf or Lb. These findings suggested that the proteome of Rf and Lb was severely reduced but has a low number of new proteins not found in other groups. The core proteome of Rf and Lb comprised a subset of proteins that were found in the other species, plus a few proteins that, to date, have only been recorded in Rf (53 proteins) or Lb (113 proteins), and which have remained undetected in either LTB or FL. In contrast, FL evolved 1282 unique proteins, and LTB evolved 1652 unique proteins (in a total of 15 species). Many of the proteins found in the genera *Spirochaeta*, *Sphaerochaeta*, *Brachyspira*, *Leptospira*, and *Treponema* were absent in Rf-Lb.

The proteome reduction in Rf and Lb affected the relevant BPs. The Rf and Lb groups had 1577 and 1627 unique proteins-processes, respectively. In contrast, the FL group had 6134 and LTB had 7805 unique proteins-processes. These values represented unique combinations of proteins and processes, and therefore, they were not affected by the number of species in each group. The most significant differences between groups were observed in carbohydrate metabolism, DNA hydrolysis, redox, translation, and transport. Regarding carbohydrate metabolism, the FL species included several proteins categorized as enzymes, including amylase, galactosidase, glucosidase, and mannosidase, and a large array of glycoside-hydrolases. These proteins were absent in all parasitic groups (i.e., LTB, Rf, and Lb). Regarding DNA hydrolysis, Rf-Lb had several polymerases and restriction endonucleases that were unique to this group. In addition, all the species in Rf-Lb lacked many reductases and dehydrogenases, in addition to proteins in the short-chain dehydrogenase/reductase family, which are a group of a NAD- or NADP-dependent oxidoreductases. However, a neutrophil-activating protein was unique to the Rf-Lb group. Different types of superoxide dismutases and thioredoxins were found in Rf and Lb, but not in LTB and FL. This finding suggested that Rf and Lb might possess a superior ability to handle cell damage produced by oxygen metabolism. In this context, it is important to note that only three ABC-type transporters were present in the Rf-Lb group, including a putrescine and spermidine transporter, an ABC transporter permease, and a ribose/galactose-dependent permease. All these proteins were present in every species of the Rf-Lb groups and absent in all species of LTB and FL groups. Several ABC-type transporters were detected in both FL and LTB species. These groups shared a variable number of proteins among the 73 different clusters of ABC-transporters. However, glutamate transporters and K^+^ transport proteins were found only in the Rf-Lb group. In addition, a sodium-dependent/pantothenate symporter was unique to the Rf-Lb group, which suggested that these species needed to import pantothenate. Interestingly, only Rf-Lb species had evolved a transporter that belonged to the family of small-conductance mechanosensitive ion channel proteins. This transporter protects cells against hypo-osmotic shock.

We built another network that comprised the same species of bacteria, but without any previous assumptions about groups. As expected, this network had a higher number of nodes and links than the previous network; however, the results with this network agreed well with other calculations in this study. We observed that every species in the Rf and Lb groups were clustered in a tight group, and species from both groups (i.e. the genus *Borrelia*) overlapped in a dense cloud. The clustering algorithms did not find an optimal solution for separating species in these two groups based on their proteome, but the groups were clearly separated from the other taxa, which also formed clusters, according to genera (Additional file 2 displays an overview of the complete network, by species-strains).

## Discussion

This study presented the largest available comparison of complete reference proteomes for selected species of the phylum Spirochaetes. We used classic methods, like dendrograms, based on presence/absence of proteins, and novel perspectives, like multivariate statistics, based on BPs, and networks, to rank protein groups according to their importance for each species. This study was not designed to explore the particular protein composition of each species; instead, we aimed to produce a large-scale comparison of different species in the phylum, including FL taxa, directly transmitted pathogens, and vector-transmitted pathogens. A large number of studies have addressed the peculiarities, functionalities, and features of the proteins characterized in this large assemblage of taxa (i.e. [17–20]); thus, we recommend that readers seek detailed descriptions of particular proteins in those reports. Here, we aimed to demonstrate that taking a higher-order view of relationships between tick-transmitted spirochetes, which involved reference proteomes, could contribute to resolving taxonomic issues, like the recent proposal that *Borrelia* should be split. Of special interest, we constructed phyloproteomic networks [17] based on the presence/absence patterns of proteins and biological processes. This method is directly linked to previous work [13] proposing that functional proteomes could be used as a reference for reconstructing phylogenetic relationships.

A wide variety of arthropod-borne pathogens place significant metabolic demands on their vectors. Previous studies have demonstrated that obligate nutritional mutualist bacteria make contributions to the metabolic pathways of the tick [21], but it remains unclear how the tick contributes to the metabolic pathways of the bacteria. This issue is of special interest for spirochaetes of the genus *Borrelia*, which have a dual life cycle, one in a vertebrate reservoir and the other in an arthropod vector. Several studies have investigated the changes in protein expression that *Borrelia* displays to evade the vertebrate immune response [20] or to develop and migrate within tick tissues [22]. Currently, *Borrelia* species are classified into two groups: the Rf and the Lb groups. Both groups have been subjected to different evolutionary pressures related to different tick vectors and different vertebrate reservoirs [15].

A previous study [1] proposed that *Borrelia* should be split into two genera (*Borrelia* for the Rf species and *Borreliella* for the Lb species), based on features of conserved signature indels and conserved signature proteins. This argument was debated [8, 23] with detailed assessments of the probable unreliabilities of that division. In contrast, a detailed study that supported the original report was published [24]. A review [25] studied other details of the microbiological features of both groups of species. This debate has persisted. For example, in UniProt, the data for *B. finlandensis* have been included as “*Borreliella*”, despite the lack of complete acceptance of a split by the scientific community.

This study demonstrated that, first, although *Borrelia* spp. have evolved a few new proteins that are unique to the Rf and Lb groups, these innovations numbered well below the number of proteins evolved by other species of parasitic spirochaetes, despite the small number of species included (higher variability would be expected in groups with more species). Second, every type of evaluation, including multivariate statistics, dendrograms, and networks, has demonstrated that there is no reliable method for separating the species in Rf and Lb into two coherent genera. In the present comparative metaproteomics study, we did not find any reliable argument that could support the separation of these species into two genera. We propose that the differences found in previous reports [1, 24] reflected the different evolutionary pressures from different groups of ticks and reservoirs that have acted on both groups of species [15]. The current framework for the evolution of *Borrelia* stands on the widely accepted hypothesis of the evolution of the ticks, this is the primordial tick assemblage carried the primitive stock of *Borrelia* (around 200 Mya), most probably derived from a bacterial symbiont of the ticks. The split of the ticks into two families, Argasidae and Ixodidae, presumably split the primitive Rf group into both families of ticks [15]. Further speciation and dissemination events of Ixodidae produced the speciation of the Lb group of *Borrelia*, following the separation of the primigenial land masses, allowing Ixodidae to spread and specialize to very diverse hosts and environmental conditions [15]. The reptiles-associated species of *Borrelia* are not yet well characterized to elaborate about their relationships with other groups of *Borrelia*, but they show Rf-related genomes with unique adaptations like those observed in the Lb group [26]. Except for *B*. *tachyglossi*, all members of this group are associated with reptile hosts. Phylogenetic reconstructions demonstrated that reptile and echidna-associated *Borrelia* species form an independent lineage that shares a common ancestor with Rf *Borrelia* [26].

This study had several limitations. The main limitation was that the annotations of the reference proteomes was incomplete. This is something that may improve in the future, as our knowledge on the function of ‘uncharacterized’ proteins expands, and will most probably provide additional support for the findings of this study. On the other hand, it is well-known that *Borrelia* species can exchange genes via the lateral transfer of plasmids, and that some of these plasmids may be lost after the bacteria are cultivated. Plasmids in *B. burgdorferi* s.l. appear to be dynamic [14]. Some plasmids were reported to be lost in cultures and/or the freeze/thaw cycles of strains [14, 27, 28]. Moreover, the insertion of plasmids into the main linear chromosome has been reported in *Borrelia* [28, 29]. These features could have led to a lack of information in the present study. This limitation was unavoidable; however, the proteomes included in this study represented the reference proteomes for each species and strain. The inclusion of reference proteomes provided the most complete knowledge currently available about the proteins of the phylum Spirochaetes. In addition, we argue that, given the large number of proteins analyzed, the lack of a few plasmid-transmitted proteins would not invalidate our results.

We propose that the conserved signature indels and conserved signature proteins observed in Rf and Lb species arose as a consequence of different evolutionary pressures derived from the peculiar lifestyles of species in both groups. In fact, the species of the *Borrelia* genus could be considered a group of dispersed genotypes with a phylogeographic structure [15]. These genotypes might be maintained through various evolutionary processes, including the ancestral polymorphism, balancing selections, adaptations to local environmental conditions, and phenotypic plasticity. Thus, the evolutionary structure of the *Borrelia* genome might be explained by the evolution of ticks, the separation of land masses, and the ecological traits that impacted the biology of these bacteria [15]. We hypothesize that the two groups of spirochaetes evolved from a stock of primitive species that colonized argasid ticks, then split into two groups of species, according to the evolution of their vectors, and these events caused differences in their proteomes. However, every test carried out on the proteomes of these bacteria have not provided evidence of sufficient differences to support a generic split, in the context of the phylum Spirochaetes. This study does not support the split of *Borrelia* + *Borreliella* as claimed in reference [24] providing enough comparative information based on more than 40,000 proteins.

## Conclusions

The proposal to separate species of the family Borreliaceae, based on the presence of conserved signature proteins, was not supported by a metaproteomics analyses. We performed dendrograms of the presence/absence of proteins, multivariate statistics based on BPs, and phyloproteomic network approaches. None of our results supported a split of the *Borrelia* genus into two genera. In fact, our results indicated that a coherent hypothetical split of *Borrelia* into two genera would require splitting most species in *Leptospira*, *Treponema*, *Brachyspira*, *Spirochaeta*, *Sphaerocheata*, and *Sediminispirochaeta* into separate genera. These splits would be unreliable, according to the many microbiological data known for these genera.

In addition, we demonstrated a large reduction in the proteome and a low number of proteins exclusive of *Borrelia*. *Borrelia* has adapted to both a vertebrate reservoir and a tick vector; this peculiar lifestyle imposes drastic conditions on its adaptation, which impacts the cell machinery of the species. Interestingly, our phyloproteomic network analysis validated the notion that most prominent proteins of *Borrelia* were shared with FL and LTB species. Thus, the proteome of the *Borrelia* genus is a subset of the other species in the phylum, with a few new proteins that evolved separately in Rf and Lb, due to their different life style traits, which affected their evolution.

## Methods

### Purpose

We performed a comparative metaproteomics analysis between several species of the phylum, Spirochaetes, including FL species, parasitic species transmitted directly without a vector, and species of the genus *Borrelia* known to have a dual lifestyle involving arthropod vectors and vertebrate hosts. We performed the analysis with the complete annotated proteomes of the selected species, including proteins of the associated BPs that were encoded in both the chromosome and the plasmids. We performed this proteome comparison to determine which proteins and/or BPs had been lost or gained during the evolution of the spirochetes included in the analysis. We investigated whether proteome differences between species of the family Borreliaceae could justify a split in the genus *Borrelia* into two genera, *Borrelia* and *Borreliella* [1]. In this functional approach, we addressed in toto proteome comparisons (i.e., metaproteomics) and purposely ignored differences in nucleotide and/or amino acid sequences. We acknowledged that this approach would not detect subtle changes in amino acidic composition or underlying genetic changes. Instead, it relied on a strong evolutionary signal based on the presence/absence of proteins and BPs among the species.

### Selected species and proteomes of reference

We did not intend to include all known species of Spirochaetes in the analysis. Instead, we selected representative species that provided a global view of the diversity of the phylum. Furthermore, only complete and well-annotated proteomes were included in analyses. The UniProt server was searched to acquire the complete proteomes (including plasmids) of 34 species and 8 strains of the genera *Sediminispirochaeta* (1 species), *Spirochaeta* (3 species), *Sphaerochaeta* (3 species), *Treponema* (3 species), *Leptospira* (3 species), *Brachyspira* (2 species), and *Borrelia-Borreliella* (19 species with several strains, which comprised 27 combinations of species-strains for *Borrelia-Borreliella*)*.* The genera *Spirochaeta* and *Sphaerochaeta* represented the FL species; the LTB genera (*Leptospira*, *Treponema*, and *Brachyspira*) represented the pathogenic species transmitted without vectors. Species in the family Borreliaceae included the classic groups of Rf species and Lb species. In most cases, the selected proteomes represented the ‘reference proteomes’ for the species (see Table 1). For some taxa, several strains of the same species were included to check for internal inconsistencies in the data.

### Protein annotation and nomenclature

Complete proteomes were obtained from UniProt [30]. General annotations were obtained from the GO Consortium website [31]. The standard name of each protein was obtained with the online tool provided by InterProt. This service also provides the “group” to which the protein belongs (“cluster”, in the terminology of InterProt) and links the protein to the major categories of GO. We used the labels of the protein clusters in further calculations. Proteins that were labeled “uncharacterized”, “hypothetical”, or “fragment” were removed from the analyses, because they represented orthologs that had been not fully characterized (these are included in Additional file 1 to ensure a complete set of raw data). UniProt links the proteins to the three major categories of annotations in the GO system, namely ‘cell compartment’, ‘molecular function’, and ‘biological process’ (BP). We only used the annotations for BPs. The list of species, with details on each strain, the group of species, the number of proteins, and the ID value of each organism and proteome obtained with the UniProt online tool are listed in Table 1.

### Data curation

Each protein could be involved in one or several BPs, because annotations were obtained from different online services linked to UniProt. One function of the manual curation of the UniProt protein database was the harmonization of annotations. However, a mass download, as in the present study, might retrieve redundant annotations from the different linked databases. Therefore, the first step was to assign the complete set of BPs to each protein, without redundancies from the different databases. Subsequently, a list of unique proteins and BPs was obtained for each of the 41 proteomes included in this study. This produced a total of 924 categories (Additional file 1), which summarized the BPs of 42,489 proteins. The list of proteins involved in each BP, grouped by species of groups of taxa, is available in Additional files 3 and 4, respectively.

### Calculation of shared proteins and BPs, dissimilarity index, and detrended canonical analysis

Each unambiguously annotated BP was entered as either ‘present’ or ‘absent’ for each species. We built a checkerboard for the presence/absence of BPs in each species-strain to perform a Detrended Canonical Analysis (DCA). We used functions available in the package ‘vegan’, version 2.5–2 [32] for the R programming environment [33]. This procedure detected unambiguous associations among the selected species of the phylum, based on the presence/absence pattern of BPs for each taxa. After reducing the variables to two axes, the similarity of the taxa involved could be compared, based on their positions (Euclidean distance) on the coordinates of the two axes of variability. Further comparative analyses were performed only for the 10 most prominent BPs, ranked according to the number of proteins involved. We investigated the total number of proteins involved in each of the 10 BPs, and how they were shared among the groups of species (FL, LTB, Rf, Lb). We constructed Venn diagrams to show how the proteins were shared, and we evaluated the Jaccard’s ID (index of dissimilarity) among the groups of species for each BP, using also the package ‘vegan [32] for R [33].

The phylogenetic distance between pairs of species/strains was calculated according to the number of shared proteins in the complete proteome, as follows. Each unambiguously identified protein was entered as either ‘present’ or ‘absent’ for each species. The number of copies of a given protein within the species was not included. Gene copy number is proportional to the genome size, an effect we purposely excluded from our analysis, with the assumption that the presence of at least one copy of the protein will ensure the presence of the BP represented by that protein. Subsequently, a large matrix (i.e., checkerboard) was used to calculate the ID. We plotted the values of the ID in a heat map; we also used the inverse of the ID among species to build a phylogenetic tree, based on patterns of the presence/absence of proteins.

### Construction of phyloproteomic networks

We used networks to reconstruct evolutionary processes that were not tree-like in nature [17]. The application of networks to proteomic data enabled the visualization of evolutionary events and taxonomic associations between taxa, proteins, and BPs, which could not be represented with a bifurcating phylogenetic tree. Networks relate nodes (taxa, proteins, BPs) through links (species with proteins that share BPs) and provide the relative importance of each node in the resulting network. Current applications of this methodology include functional comparisons across the three domains of life [34], prokaryotes [35], bacterial plasmids [36], and bacteriophages [37]. Networks allow the calculation of several indexes to evaluate the relative importance of each node and the strengths of the links within the network. One of these indexes is called the Betweenness Centrality (BNC), which measures the shortest path between nodes. Thus, the BNC measures the importance of each node in the “flow of information” through the network. The use of the BNC as an indicator of the prominence of a node was previously proposed for other systems [38].

We built an undirected network, based on the proteins recorded for every species-strain. This network was constructed based on the proteins shared between groups of species in pairwise combinations (Rf, Lb, FL, LTB). With this approach, the nodes in the network were species, proteins, and the BPs in which the proteins were involved. The edges linking these nodes corresponded to BPs shared by proteins detected in the selected taxa. For example, an organism A, which produced protein B, which was involved in process C, would be displayed with a connection, as follows: A➔B➔C. Thus, this network considered the phylogenetic context of species from a different perspective by rendering meaningful functional comparisons. All calculations were performed for each pairwise combination of taxa. Clusters of proteins and species were calculated with the Louvain algorithm [39]. Clusters were defined as groups of nodes (groups of species or proteins) that interacted more among each other than with the other nodes [38]. In this context, clusters reflected the proteins that were shared by a group of taxa, and its BNC indicated “how important” these proteins were in the complete proteome of the species, relative to the presence/absence of other proteins. A second network was computed with the complete set of species (i.e., without previous assumptions about groups), and the BPs were used as nodes, instead of proteins. The network building process was unchanged, but in this case, the links were based on the species and the BPs.

## Additional files

Additional file 1: All the biological processes and the associated number of proteins involved, according to taxa. It is a pivot table that links the Biological processes in a hierarchical structure (first column). Each additional column lists the name of the species and strains and the number of proteins recorded for each species in such particular category. (XLSX 505 kb)

Additional file 2: A summarized network of proteins found in the species of the Spirochaetes phylum. Only the bacterial species are labeled to improve readability. Each circle represents either a protein (unlabeled) or a species (labeled). Each cluster of species and their associated proteins are color-coded, and links indicate proteins that are shared by other groups. The colors are random and are intended only to separate the clusters. The size of the circle indicates the betweenness centrality of the node in the network; this property is related to its centrality (the number of links that pass through the node) in the groups of Spirochaetes. (XLSX 142 kb)

Additional file 3: The top ten biological processes and their annotated proteins, separated by species and strains, including the number of times the protein was recorded in each species. It is a pivot table that links the Biological processes in a hierarchical structure and the proteins annotated to perform such process (first column). Each additional column lists the name of the species and strains and the number of proteins recorded for each species. (XLSX 103 kb)

Additional file 4: The top ten biological processes and their annotated proteins, separated by groups of species (FL: free-live; Rf: relapsing fever; Lb: Lyme borreliosis; LTB: genera *Leptospira*, *Treponema* and *Brachyspira*), including the number of times the protein was recorded in each species. It is a pivot table that links the Biological processes in a hierarchical structure and the proteins annotated to perform such process (first column). Each additional column lists the name of the species and strains and the number of proteins recorded for each group of species. (PDF 830 kb)

## Abbreviations

BNC: Betweenness Centrality
BP: Biological process (plural, BPs, biological processes)
DCA: Detrended Canonical Analysis
FL: Free-life
ID: Jaccard’s index of dissimilarity
Lb: *Borrelia* spp., Lyme borreliosis group
LTB: genera *Leptospira*, *Treponema* and *Brachyspira*
Rf: *Borrelia* spp., relapsing fever group
