# Supplementary figures and images for: Phyloproteomic and functional analyses do not support a split in the genus Borrelia (phylum Spirochaetes)
Source: BMC Evol Biol. 2019 Feb 13;19:54. doi: 10.1186/s12862-019-1379-2 (PMC6375133; doi:10.1186/s12862-019-1379-2)

Additional file 4

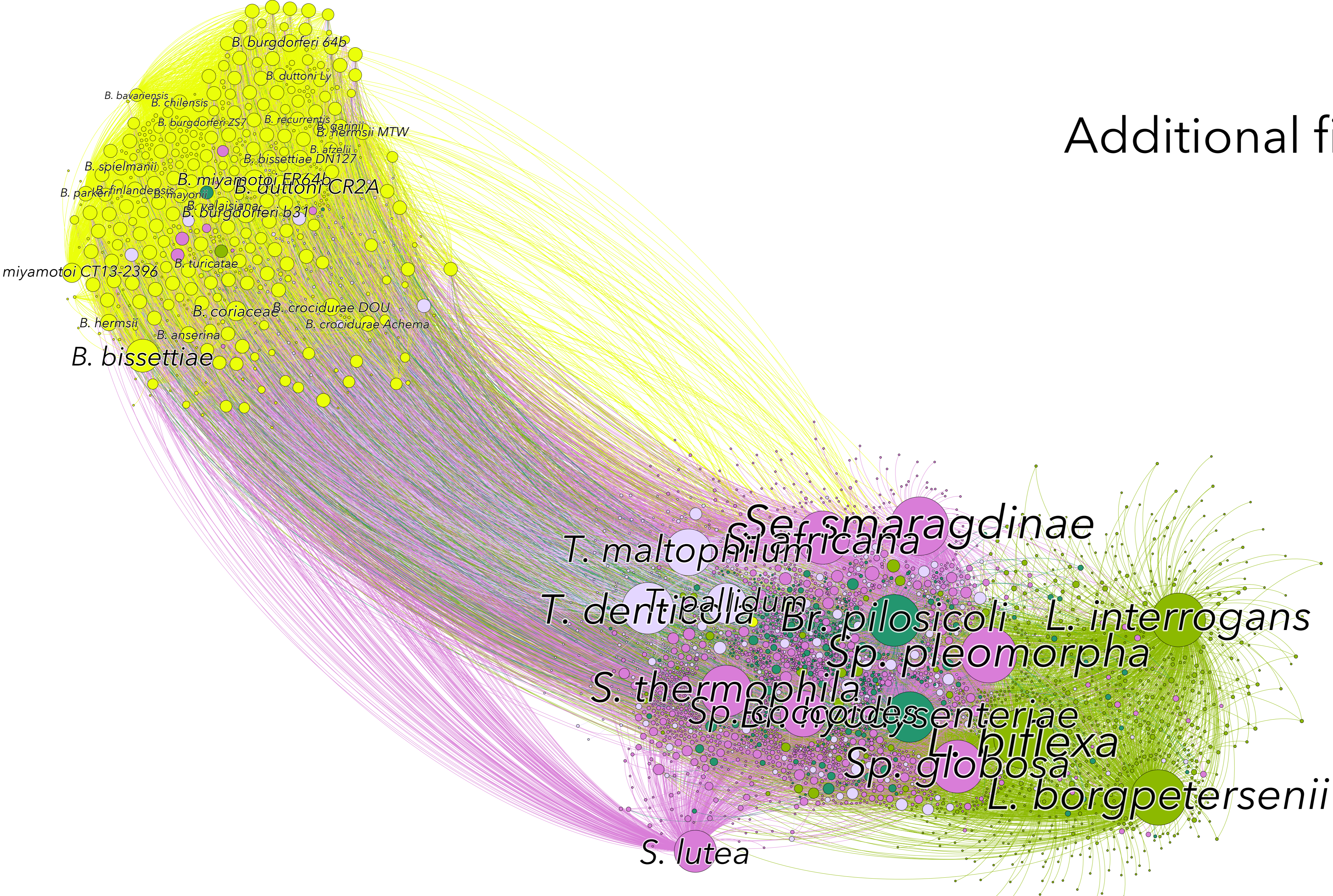

Supplement: Supplementary file 4 — The top ten biological processes and their annotated proteins, separated by groups of species (FL: free-live; Rf: relapsing fever; Lb: Lyme borreliosis; LTB: genera Leptospira, Treponema and Brachyspira), including the number of times the protein was recorded in each species. It is a pivot table that links the Biological processes in a hierarchical structure and the proteins annotated to perform such process (first column). Each additional column lists the name of the species and strains and the number of proteins recorded for each group of species. (PDF 830 kb) [file 12862_2019_1379_MOESM4_ESM.pdf]
